# The negative regulators of Wnt pathway—*DACH1*, *DKK1*, and *WIF1* are methylated in oral and oropharyngeal cancer and *WIF1* methylation predicts shorter survival

**DOI:** 10.1007/s13277-014-2913-x

**Published:** 2014-12-07

**Authors:** Jarosław Paluszczak, Joanna Sarbak, Magdalena Kostrzewska-Poczekaj, Katarzyna Kiwerska, Małgorzata Jarmuż-Szymczak, Reidar Grenman, Daniela Mielcarek-Kuchta, Wanda Baer-Dubowska

**Affiliations:** 1 Department of Pharmaceutical Biochemistry, Poznań University of Medical Sciences, ul. Święcickiego 4, 60-781 Poznań, Poland; 2 Department of Mutagenesis, Institute of Human Genetics, Polish Academy of Sciences, Poznań, Poland; 3 Department of Otorhinolaryngology, Head and Neck Surgery, Turku University Central Hospital and Turku University, Turku, Finland; 4 Department of Medical Biochemistry, Turku University Central Hospital and Turku University, Turku, Finland; 5 Department of Otolaryngology and Clinical Oncology, Poznań University of Medical Sciences, Poznań, Poland

**Keywords:** Oral cancer, DNA methylation, WIF1, Wnt pathway

## Abstract

The deregulation of Wnt signaling has recently emerged as one of the drivers of head and neck cancers. This is frequently related to the methylation of several antagonists of this pathway. This study aimed at the assessment of the profile of methylation of Wnt pathway antagonists and the determination of the prognostic value of the methylation of selected genes in oral carcinomas. The methylation of *DACH1*, *DKK1*, *LKB1*, *PPP2R2B*, *RUNX3*, *SFRP2*, and *WIF-1* was analyzed in 16 oral squamous cell carcinoma cell lines using the methylation-specific polymerase chain reaction. The methylation of selected genes was further analyzed in tumor sections from 43 primary oral carcinoma patients. The analysis of oral carcinoma cell lines showed very frequent methylation of *SFRP2* and *WIF-1* and also a less frequent methylation of *DACH1* and *DKK1*. On the other hand, *RUNX3* was methylated only in one cell line, while *LKB1* and *PPP2R2B* were not methylated in any of the cell lines*.* The biallelic methylation of *DKK1* correlated with the low level of expression of this gene. Further evaluation of the methylation of *DACH1*, *DKK1*, and *WIF1* in a clinical patient group confirmed the frequent methylation of *WIF1* and intermediate or low frequency of methylation of *DACH1* or *DKK1*, respectively. Importantly, the methylation of *WIF-1* correlated with shorter survival in oral cancer patients. Overall, the methylation of the antagonists of Wnt pathway is frequently detected in oral squamous cell carcinomas. The methylation of *WIF1* may be considered a prognostic marker in oral cancers.

## Introduction

Oral and oropharyngeal squamous cell carcinoma (OSCC) is a common cancer of the head and neck region, and it ranks as the eighth most prevalent cancer among males in the USA [1]. The overall 5-year survival rates are around 50–60 %, and despite the growing understanding of the molecular pathogenesis of head and neck squamous cell carcinomas (HNSCC), therapy outcomes remain unaltered what is explained by a relatively frequent rate of tumor relapse or the appearance of metastases [2]. The improvement of treatment outcomes may be facilitated by the discovery of new diagnostic markers. Among molecular markers, epigenetic biomarkers based on the analysis of DNA methylation profiles have already proven to be useful in both the detection and the prognostication of cancer [3].

The Wnt/β-catenin signaling pathway serves for the regulation of cell proliferation, migration, and apoptosis. When the pathway is in the resting state, the chief protein that serves for signal transduction–β-catenin is phosphorylated by an inhibitory complex which comprises of glycogen synthase kinase 3β (GSK-3β), casein kinase 1α, adenomatous polyposis coli (APC), and Axin. Phosphorylated β-catenin is immediately degraded via the ubiquitin-proteasome pathway, and thus, the cytoplasmic pool of β-catenin is kept at a very low level. During pathway activation, the binding of Wnt ligands to Frizzled receptors activates Dishevelled, which inhibits the activity of GSK-3β and blocks the degradation of β-catenin. This enables the accumulation of β-catenin in the cytoplasm and its subsequent translocation to the nucleus where it binds the TCF/LEF family of transcription factors and stimulates the expression of genes which enhance cell cycle and cell migration, such as *CCND1*, *MYC*, *MMP-7*, or *survivin* [4].

Mutations in the genes coding for proteins in the Wnt pathway are rare in HNSCC, and thus, this pathway was not believed to be significant for the pathogenesis of head and neck carcinomas [5–8]. However, recent investigations indicate that the hyperactivation of the pathway may result from a different mechanism which is based on the aberrant hypermethylation of the negative regulators of Wnt pathway. The genes which encode the extracellular antagonists of Wnt ligands or Frizzled/LRP receptors, such as *SFRP1-5*, *WIF1*, or *DKK1-3*, are frequently silenced in HNSCC cells through the methylation of their promoter regions. Also, the function of the intracellular negative Wnt regulators such as *DACH1*, *PPP2R2B*, or *RUNX3* may be lost due to the hypermethylation of their gene promoters [7, 9–11]. It has been also reported that the expression of Wnt ligands, Frizzled receptors, and Dishevelled as well as β-catenin is increased in head and neck (HN) carcinoma cells [7, 12–15]. Moreover, the appearance of nuclear β-catenin and the enhancement of the expression of β-catenin target genes, such as *MYC*, *CCND1*, *MMP-7*, or *survivin* are often observed in HNSCC cancer cells. Importantly, such changes seem to be cancer-specific since nuclear β-catenin was not detected in normal oral mucosa in contrast to oral leukoplakia. Additionally, dysplastic leukoplakia showed stronger nuclear accumulation of β-catenin than non-dysplastic leukoplakia [16]. Another study showed that the upregulated proliferation of basaloid cells in oral epithelial dysplasia may be caused by enhanced Wnt signaling [17]. Accumulating evidence suggests that the activation of Wnt signaling escalates with the progression of head and neck cancers. Elevated expression of β-catenin correlated with shorter survival of patients with oral carcinomas [13]. In line with this observation, the knockdown of β-catenin reduced the growth of HN cancer cells and tumors [18, 19]. Moreover, the expression of β-catenin was higher in poorly differentiated tumors than in moderate and well-differentiated tumors. Indeed, β-catenin may inhibit the differentiation of keratinocytes through the upregulation of Myc [13]. Another study showed that the hyperactivation of β-catenin affects cell morphology and cell adhesion leading to the higher capacity of cells for invasion and migration driven i.a. by β-catenin-induced activation of MMP-7 expression [20]. This indicates that the cytoplasmic and nuclear accumulation of β-catenin contributes to epithelial to mesenchymal transition in HN carcinoma cells and, thus, may be significantly associated with local recurrence and lymph node metastasis. All these facts underscore the biological importance of the aberrations in Wnt signaling in the pathogenesis of HNSCC and indicate the possible prognostic significance of changes in Wnt pathway activity in disease diagnostics.

The aim of the present study was to assess the frequency of gene promoter methylation of Wnt pathway antagonists (*DACH1*, *DKK1*, *LKB1*, *PPP2R2B*, *RUNX3*, *SFRP2*, and *WIF-1*) in oropharyngeal squamous cell carcinoma cell lines and establish the diagnostic potential of the most promising genes in respect to correlation with important clinicopathologic data such as tumor recurrence or disease-free survival in a group of oropharyngeal cancer patients. We indicate that the analysis of the methylation of the promoter region of *WIF-1* constitutes the most promising prognostic biomarker in OSCC patients.

## Materials and methods

### Oral cancer cell lines

Sixteen cell lines derived from oral squamous cell carcinoma patients at the University of Turku, Finland were used in this study. Table 1 presents the characteristics of the original material taken to establish each of the cell lines.

**Table 1:** The characteristics of the patients and tumors that were taken to establish the analyzed laryngeal squamous cell carcinoma cell lines

Cell line	Sex	Age (years)	Primary tumor location	TNM classification	Specimen site	Type of lesion	Histological grade
UT-SCC-10	M	62	SCC linguae	T_1_N_0_M_0_	Tongue	pri	G2
UT-SCC-16A	F	77	SCC linguae	T_3_N_0_M_0_	Tongue	pri	G3
UT-SCC-16B	F	77	SCC linguae	T_3_N_0_M_0_	Neck	met	G3
UT-SCC-20A	F	58	Floor of mouth	T_1_N_0_M_0_	Floor of mouth	pri (per)	G2
UT-SCC-20B	F	58	Floor of mouth		Floor of mouth	resid	G2
UT-SCC-24A	M	41	SCC linguae	T_2_N_0_M_0_	Tongue	pri	G2
UT-SCC-24B	M	41	SCC linguae		Neck	met (per)	G2
UT-SCC-28	F	58	Floor of mouth	T_2_N_0_M_0_	Floor of mouth	pri (per)	G1
UT-SCC-36	M	46	Floor of mouth	T_4_N_1_M_0_	Floor of mouth	pri	G3
UT-SCC-45	M	76	Floor of mouth	T_3_N_1_M_0_	Floor of mouth	pri	G3
UT-SCC-47	M	78	Floor of mouth	T_2_N_0_M_0_	Floor of mouth	pri	G3
UT-SCC-56	M	62	Floor of mouth	T_x_N_2_M_0_	Floor of mouth	rec	G2-G3
UT-SCC-85	M	55	SCC marginum linguae	T_3_N_0_M_0_	Tongue-floor of mouth	rec	G2
UT-SCC-90	M	35	SCC linguae	T_1_N_0_M_0_	Floor of mouth	rec/met	G2
UT-SCC-100	M	70	SCC gingiva mandibularis	rT_3_	Mucosae bucchae	rec	G3
UT-SCC-104	M	80	SCC plicae ventricularis	T_1_N_2A_M_0_	Neck	met	G2

*M* male, *F* female, *SCC* squamous cell carcinoma, *pri* primary tumor, *per* persistent, *met* metastasis, *rec* recurrent tumor

### Patients

Forty-three patients with primary oral squamous cell carcinoma who were primarily treated surgically at the Department of Otolaryngology and Clinical Oncology, Poznań University of Medical Sciences between 2008 and 2012 were enrolled for the study. Clinical data are collected in Table 2. All the samples underwent histopathological examination and were verified to contain at least 80 % of cancer cells. The follow-up observation in most cases covered at least 3 years following surgery. Thirteen patients died of cancer during follow-up. Seven patients were lost from ongoing observation after 3 years of follow-up. Moreover, only incomplete observations were available for another six patients whose follow-up observation lasted for less than 3 years (2–24 months). Follow-up information was lacking in the case of two patients. The study was approved by the Poznan University of Medical Sciences ethics committee (approval no. 1199/08).

**Table 2:** Patient and tumor characteristics

Age (years)	Range	29–85
	Mean	58
Sex	male	40
	female	3
T classification	T1	4
	T2	19
	T3	10
	T4	10
N classification	N0	19
	N1	13
	N2	10
	N3	1
Histological grade	G1	6
	G2	33
	G3	4
Tumor localization	Tonsils	13
	Tongue	7
	Tongue and floor of the mouth	14
	Palate	2
	Lips	1
	Base of the tongue and tonsils	6

### Methylation-specific PCR

DNA was extracted from samples using a standard phenol/chloroform protocol. The methylation status of *DACH1*, *DKK1*, *LKB1*, *PPP2R2B*, *RUNX3*, *SFRP2*, and *WIF-1* was assessed using the methylation-specific polymerase chain reaction (MSP) [21], as previously described [9]. The primers and reaction conditions for MSP were chosen based on previously published data [7, 22–25]. All the primers were obtained from Oligo.pl (Warsaw, Poland). DNA extracted from the lymphocytes of healthy blood donors and a completely methylated human DNA (Fermentas, Burlington, Canada) were used as the negative and positive MSP control, respectively. Genomic DNA samples derived from early passage primary culture of human tracheal epithelial cells and human oral keratinocytes (ScienCell Research Laboratories, Carlsbad, CA, USA) were used as normal controls for comparison. Amplification products were resolved on 2 % agarose gels and visualized under UV light illumination.

### Quantitative PCR

Total RNA was extracted by standard phenol/guanidine thiocyanate extraction and subjected to reverse transcription using the RevertAid Kit (Fermentas). Quantitative real-time PCR was performed using the HOT FIREPol Eva Green qPCR Mix (Solis BioDyne, Tartu, Estonia) and a Chromo4 thermal cycler (BioRad Laboratories, Hercules, CA, USA). The amplification protocol started with a 15-min enzyme activation at 95 °C, followed by 40 cycles of 95 °C for 20 s, 56 °C for 20 s, and 72 °C for 40 s and the final elongation at 72 °C for 5 min. The melting curve analysis was used for the verification of the lack of non-specific products. Measurements were normalized for the expression of *TATA box binding protein* (TBP). Primer sequences are listed in Table 3.

**Table 3:** The sequence of starters used in real-time PCR reactions

Primer	Sequence	Product size
*TBP* forward	5′GGCACCACTCCACTGTATC	183 bp
*TBP* reverse	5′GGGATTATATTCGGCGTTTCG	
*DACH1* forward	5′CAAGTGTCGGACTGGAAC	172 bp
*DACH1* reverse	5′GATGTCTCAACTCTGGATGG	
*DKK1* forward	5′GGAAACCATCACTGAAAGC	154 bp
*DKK1* reverse	5′AGCACAACACAATCCTGAG	
*WIF1* forward	5′ATTCCTGTCAATATCCATTCC	163 bp
*WIF1* reverse	5′CAACTGATGCCTTGTGAG	

### Statistical analysis

The correlation between clinicopathologic features and gene methylation was assessed with chi-square or Fisher’s exact test and F-Cox test (*p* ≤ 0.05) using STATISTICA 10.

## Results

In the first stage, 16 oral squamous cell carcinoma cell lines were screened for the methylation of *DACH1*, *DKK1*, *LKB1*, *PPP2R2B*, *RUNX3*, *SFRP2*, and *WIF-1* using methylation-specific PCR. We detected very frequent methylation of *WIF1* (93.7 %) and *SFRP2* (81.2 %) and moderately frequent methylation of *DKK1* (37.5 %) and *DACH1* (31.2 %). *RUNX3* was found methylated in only one cell line (6.25 %), and the other genes (*LKB1* and *PPP2R2B*) were not methylated in any of the cell lines. In order to assess whether the observed profile of methylation changes is specific for cancer cells, we analyzed the status of methylation of all the genes in normal cells of the upper aero-digestive tract—tracheal epithelial cells and oral keratinocytes. None of the genes was methylated in the genomic DNA derived from normal cells, except for *SFRP2*, which showed partial methylation in tracheal epithelial cells. Therefore, *SFRP2* was excluded from further analysis.

**Fig. 1:**
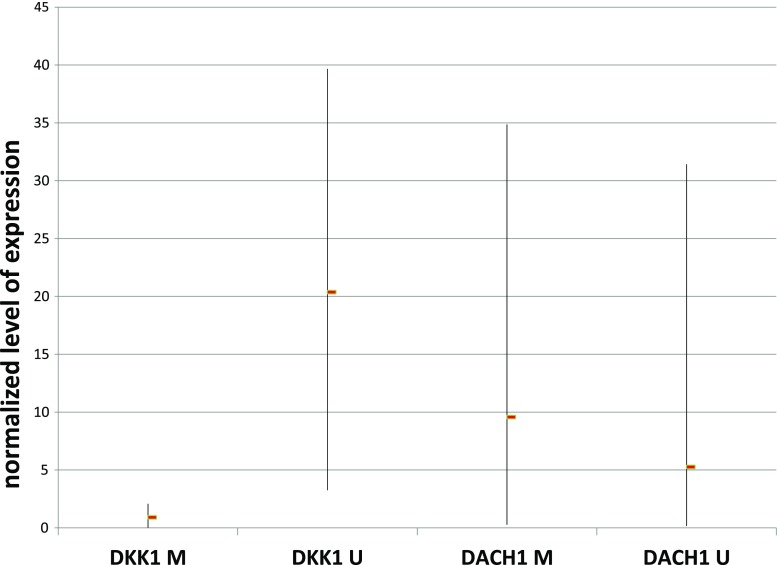
The relationship between the promoter methylation of *DACH1* and *DKK1* and normalized gene expression (transcript level). Normalized level of transcript is expressed as the ratio calculated from the formula: Ratio (reference gene/target gene) = 2 ^Ct (reference) − Ct (target)^, where *TBP* is the reference gene and either *DACH1* or *DKK1* is the target gene. *Each bar* represents the range of results obtained for all the samples in the respective groups with the mean value marked with the *rectangle. M* gene promoter methylation, *U* complete lack of gene promoter methylation

Next, we analyzed whether there is any association between the status of methylation of gene promoters of *DACH1*, *DKK1*, and *WIF-1* and their level of expression in the cell lines (Fig. 1). In the case of *DACH1*, we found similar levels of the transcript in all the cell lines, irrespective of gene methylation status. This was however not surprising since, when present, usually partial (probably monoallelic) methylation was observed for this gene. On the other hand, complete gene silencing (methylation of both alleles) was usually observed in the cell lines positive for *DKK1* methylation. This biallelic methylation of *DKK1* was accompanied by low transcript level when compared with cell lines showing the lack of methylation of this gene. As expected, the expression of *WIF1* was almost undetectable in the analyzed cell lines due to heavy methylation of the gene promoter.

Based on these results, we identified *DACH1*, *DKK1*, and *WIF-1* as potential epimarkers in OSCC and further analyzed their methylation in a group of oral squamous cell carcinoma patients. We detected frequent methylation of *WIF-1* (63.9 %) and moderately frequent methylation of *DACH1* (35.9 %). On the other hand, only rare cases of methylation of *DKK1* (7.1 %) were observed. One fifth of the patients did not show the methylation of any of the tested genes. Usually, only one of the genes was methylated in the patient’s tumor DNA sample. Another one fifth of the patients showed the methylation of two genes (mostly the co-methylation of *DACH1* and *WIF1*). Only one patient showed the methylation of all three genes.

**Fig. 2:**
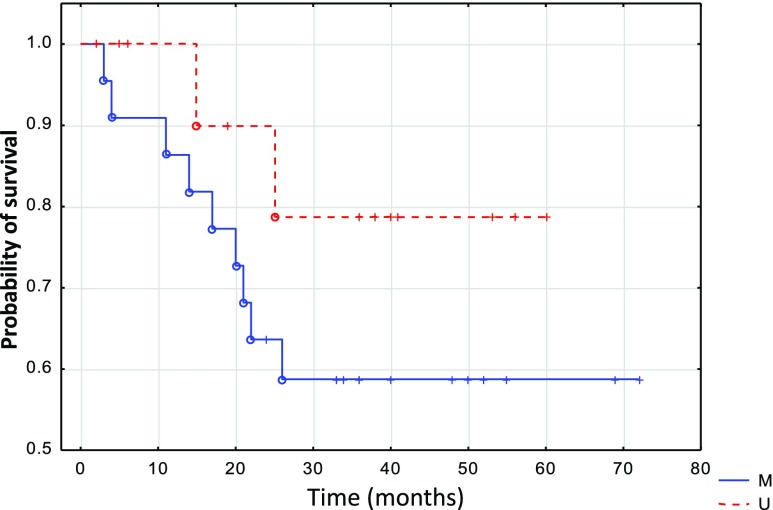
Disease-free survival estimated by *WIF-1* methylation in primary oropharyngeal tumor patients. *M* patients showing *WIF-1* methylation, *U* patients showing lack of *WIF1* methylation

The methylation of the analyzed genes did not correlate with either tumor stage (T), nodal involvement (N), tumor localization, or local recurrence. The methylation of *WIF1* significantly correlated (*p* = 0.036) with shorter survival (Fig. 2). Also, the methylation of *DKK1* correlated with histological grade (*p* = 0.002). However, both observations should be interpreted with caution because of the relatively low number of subjects enrolled in the study.

## Discussion

The number of new cases and the effectiveness of therapy of head and neck squamous cell carcinomas has remained relatively unaltered over the last decade. Mean 5-year survival rates around 60 % can be reached when surgery and/or radiotherapy are applied as standard treatment options [1]. The effectiveness is higher in patients with low-grade tumor and dramatically decreases in advanced cancer. Despite the growing understanding of the molecular changes that take place at different stages of the disease, there are still no definite markers which could be used to identify patients with increased risk of disease progression. So far, there are no approved markers of the risk of local relapse or other prognostic tests. The detection of such markers would allow the identification of patients showing worse prognosis and a higher risk of relapse and inform the necessity of application of more aggressive treatment. On the other hand, treatment options for such patients are still lacking and this gap needs to be filled in order to improve therapy outcomes in this group of patients.

The molecular aberrations in the Wnt signaling pathway have recently emerged as important drivers of HNSCC [6, 16, 17, 20]. This insight can be utilized in both the diagnostics and treatment of this group of patients. It has been reported that the activation of this pathway increases with tumor progression and, indeed, the nuclear accumulation of β-catenin correlated with shorter survival of patients with oral carcinomas [13]. Since mutations of genes in this pathway are rare in HNSCC, epigenetic changes seem to be the leading mechanism for the induction of the hyperactivation of this signaling cascade. In this regard, frequent hypermethylation of the negative regulators of this pathway (*DKK-3*, *RUNX3*, *SFRP1*, *SFRP2*, *SFRP4*, *SFRP5*, and *WIF1*) was observed in oral and oropharyngeal cancers [7, 10, 11, 26–28]. However, the diagnostic significance of these alterations remains to be elucidated. The aim of the current study was to assess the profile of methylation changes of a broader set of Wnt pathway antagonists and analyze the diagnostic significance of these potential epimarkers. In the first stage, cell lines derived from OSCC were screened for the presence of methylation of *DACH1*, *DKK-1*, *LKB1*, *PPP2R2B*, *RUNX3*, *SFRP2*, and *WIF1*. Genes which were frequently methylated in the cell lines and showed the lack of methylation in DNA samples from normal control cells were further analyzed in a group of primary OSCC patients.

The methylation of *LKB1* was not present in any of the tested cell lines. We have previously shown that this gene does not undergo methylation in laryngeal carcinomas [9]. This is in agreement with the findings that the downregulation of *LKB1* in head and neck cancers is independent of promoter methylation [29]. Similarly, *PPP2R2B* was not methylated in any of the cell lines. This differentiates oral carcinomas from laryngeal carcinomas where this gene is very frequently hypermethylated and shows prognostic significance [9]. It indicates that other mechanisms are responsible for the decrease in the activity of PP2A protein phosphatase complex which is observed in oral cancers [30].

In the case of *RUNX3*, we observed its methylation in only one cell line and thus did not analyze its methylation in primary carcinoma patients. A similar result was observed in laryngeal carcinomas [9]. Similarly, the gene was shown to be hypomethylated in tongue squamous cell carcinoma [31]. In contrast, others reported the hypermethylation of *RUNX3* in 17.8 % OSCC [28]. Moreover, it was detected in 25 % of tongue carcinomas where it correlated with tumor stage and lymph node involvement [26]. Another study showed that *RUNX3* was hypermethylated in 70 % OSCC and that the downregulation of RUNX3 protein correlated with poor differentiation [32]. Additionally, another study showed that *RUNX3* was methylated in 60 % of cases of oral dysplasia and 100 % cases of OSCC [11]. There is discrepancy concerning the actual role of *RUNX3* in HNSCC, and some authors suggest an oncogenic function for this gene [33]. This needs to be addressed in further functional studies.

Of all the antagonists of Wnt pathway, the methylation of genes encoding SFRP proteins was most frequently reported in oral carcinomas. The rate of methylation of *SFRP2* widely ranged (35–90 %) among various studies [7, 10, 11, 27]. We also detected frequent methylation (81.2 %) of this gene in OSCC cell lines. However, we observed that this was not fully cancer-specific. Similarly, the methylation of *SFRP1/2/4/5* was also present in normal control samples, although *SFRP2* showed the highest cancer specificity among the *SFRP* genes [10]. These observations suggest that although *SFRPs* are often methylated in OSCC, they might not present good diagnostic performance due to unsatisfactory specificity.

We report, for the first time, that *DACH1* undergoes methylation in oral squamous cell carcinomas. So far, the methylation of this gene was detected in colorectal carcinomas where it correlated with late tumor stage, poor differentiation, and lymph node metastasis [23]. Recently, *DACH1* emerged as an important tumor suppressor gene [34] and its frequent methylation in OSCC underscores the potential anti-cancer effects of epigenetic therapies via suppression of Wnt signaling. Indeed, it has been shown that the inhibition of Wnt pathway blocks tumor growth in head and neck cancers [18, 19].

Although the methylation of *DKK1* was infrequent in primary OSCC, it strongly correlated with histological grade. To our knowledge, the methylation of this gene was not analyzed in oral cancers although the methylation of another member of the dickkopf family of genes—*DKK3—*was previously reported [10]. However, it was found that the expression of *DKK1* correlated significantly with a low risk of regional lymph node metastasis and that its knockdown increased the cellular migration and invasiveness in oral cancer cells [35]. This suggests a potential prognostic significance of the methylation of this gene in oral carcinomas. Although no evidence in favor of this hypothesis was found in our study, this may be attributed to a relatively small cohort of patients regarding the low frequency of methylation in the group of primary OSCC patients.

In our study, *WIF1* was the most frequently methylated gene in oral carcinomas. It was previously reported to be methylated in 18 % OSCC [10] and 35 % of tongue carcinoma patients [26]. Moreover, the methylation of this gene can be considered as a marker of progression of carcinogenesis since its methylation was observed to be twice more frequent in OSCC (80 %) than in oral dysplasia (40 %) [11]. Importantly, we observed that the methylation of the gene correlated with shorter overall survival of the patients. Although such a correlation was not observed in tongue carcinomas [26], others reported a similar observation in the case of non-small cell lung cancer [36].

The results presented in this study corroborate that the epigenetic abnormalities may frequently affect Wnt signaling in oral carcinomas. Roughly, 80 % of the patients showed the methylation of at least one gene. It indicates that this pathway should be considered a possible therapeutic target in OSCC apart from the fact that the analysis of promoter methylation of genes in this cascade presents promising diagnostic possibilities.

In summary, our current study showed that *DACH1*, *DKK1*, and *WIF-1* are methylated in oral and oropharyngeal squamous cell carcinomas. Moreover, oral cancers are differentiated from laryngeal carcinomas by the lack of methylation of *PPP2R2B*. The possible prognostic significance of the methylation of *DKK1* and *WIF1* needs to be further evaluated in prospective studies.

## Abbreviations

HNSCC: Head and neck squamous cell carcinoma
OSCC: Oral squamous cell carcinoma
